# Using an internet intervention to support self-management of low back pain in primary care: protocol for a randomised controlled feasibility trial (SupportBack)

**DOI:** 10.1136/bmjopen-2015-009524

**Published:** 2015-09-23

**Authors:** Adam W A Geraghty, Rosie Stanford, Paul Little, Lisa Roberts, Nadine E Foster, Jonathan C Hill, Elaine Hay, Beth Stuart, David Turner, Lucy Yardley

**Affiliations:** 1 Department of Primary Care and Population Sciences, University of Southampton, Southampton, Hampshire, UK; 2 Department of Health Sciences, University of Southampton, Southampton, Hampshire, UK; 3 Research Institute of Primary Care and Health Sciences, Keele University, Stoke-on-Trent, UK; 4 Norwich Medical School, University of East Anglia, Norwich, UK; 5 Centre for Applications of Health Psychology, University of Southampton, Southampton, Hampshire, UK

**Keywords:** PRIMARY CARE, REHABILITATION MEDICINE

## Abstract

**Introduction:**

Low back pain (LBP) is a prevalent and costly condition. The majority of patients experiencing LBP are managed in primary care, where first-line care recommendations consist of advice to self-manage and remain active. Internet interventions present a potential means of providing patients with tailored self-management advice and evidence-based support for increasing physical activity.

**Methods/analysis:**

This protocol describes a single-blind, randomised controlled feasibility trial of an internet intervention developed to support the self-management of LBP in primary care. Patients are being randomised to 1 of 3 groups receiving either usual primary care, usual primary care with the addition of an internet intervention or an internet intervention with physiotherapist telephone support. Patients are followed up at 3 months. Primary outcomes are the feasibility of (1) the trial design/methods, (2) the delivery of the internet intervention and (3) the provision of telephone support by physiotherapists. Secondary outcomes will include exploratory analysis of estimates and variation in clinical outcomes of pain and disability, in order to inform a future main trial.

**Ethics/dissemination:**

This feasibility trial has undergone ethical scrutiny and been approved by the National Health Service (NHS) Research Ethics Committee, REC Reference 13/SC/0202. The feasibility findings will be disseminated to the research community through presentations at conferences and publication in peer review journals. Broader dissemination will come following a definitive trial.

**Trial registration number:**

ISRCTN 31034004.

**Strengths and limitations of this study**

- This protocol describes a randomised controlled feasibility trial of a novel internet intervention for primary care patients with low back pain, provided in addition to usual primary care.
- This study will explore adding physiotherapist telephone support to the internet intervention.
- Using the internet as a delivery mode for the intervention may exclude some primary care patients where access to the internet is limited.

## Introduction

Low back pain (LBP) is common; lifetime prevalence estimates range from 65% to 84%.^1^ ^2^ Each year approximately 6–9% of the UK population will visit their general practitioner (GP) with LBP, at a cost of around 251 million pounds to the National Health Service (NHS) per annum.^3^ ^4^ Although acute LBP is often reported as a relatively brief, self-limiting condition,^5^ Henschke *et al*^6^ showed that around a third of patients presenting with acute symptoms had not recovered at 12 months. In studies including all primary care consulters with LBP, up to 52% of patients had poor clinical outcome at 6 months,^7^ and Dunn *et al*^8^ showed LBP trajectories for primary care consulters appear to remain stable with 69% of patients continuing to report mild to persistently severe pain at 7-year follow-up. Where LBP becomes recurrent or persistent (estimates range widely from 2% to 56%^9^ ^10^), patients are at risk of becoming deconditioned, depressed and immobile, reducing their ability to work and leading to extensive healthcare and societal costs. With regard to disease burden, Hoy *et al*^11^ recently demonstrated that LBP caused more global disability than any other condition.

Promoting effective self-management is a key strategy in the care of patients with LBP.^12^ ^13^ Definitions of self-management vary; however, central components include monitoring of the health condition and effecting the cognitive, behavioural and emotional responses necessary to maintain quality of life, and where possible, reduce symptom-related disability.^14^ Encouraging physical activity and exercise is recommended within LBP treatment guidelines,^13^ ^15^ and physical activity is a core component of self-management programmes.^12^ Alongside physical activity, targeting psychological processes such as self-efficacy,^16^ pain perceptions, expectations and fear-avoidance beliefs may also be important in interventions for LBP.^17^ For those needing support to regain their self-efficacy, overcome common concerns and regain active lifestyles, GP referral to physiotherapy is common, with UK estimates suggesting 20% are referred to a physiotherapist in the UK.^18^ Recent research suggests caution regarding advice on the pharmacological management of symptoms;^19^ concerns have been raised about the effectiveness of paracetamol^20^ and oral non-steroidal anti-inflammatory drugs (NSAIDs) may not be suitable for patients with contraindications, particularly prevalent in older adults.^13^ This recent research highlights the important role of non-pharmacological self-management in the care of patients with LBP.

The majority of patients with LBP will be seen by GPs in primary care.^21^ GPs’ consultations with patients are typically short, and after ruling out serious spinal pathology, GPs are unlikely to have the resources or training to deliver optimal evidence-based behavioural support. Interventions delivered over the internet may be well placed to complement time-constrained GPs in providing tailored information, advice and support for self-management.

Internet interventions are structured programmes that patients engage with by using computers or mobile devices (including tablets), over a set period of time. In addition to providing tailored educational information, they can support self-management by facilitating goal setting, self-monitoring and providing behavioural/symptom-related feedback. Along with heightened interactivity, internet interventions can provide audio and video material, broadening the scope for content beyond traditional paper-based media for delivering LBP-related education and advice.^22^ Internet interventions delivered with ‘live’ health professional support, which can be minimal, have been shown to be more effective than internet interventions delivered without support.^23^ However, this is not always the case^24^ and removal of health professional time may ensure these interventions can be offered more broadly and cost-effectively, potentially increasing access.

With all technology-mediated health innovations, it is essential to remain vigilant to how access limitations might exclude certain groups of society. Nonetheless, it is important to reflect on this in light of access to traditional services. For instance, household internet access in the UK is reported to have now reached 84%,^25^ and is expected to continue to grow. These increasing figures suggest that internet interventions may in fact become more accessible than many traditional face-to-face services, where patients’ access will be restricted by factors such as availability of transport, childcare and working hours.

Previous research on internet interventions for back pain has focused almost exclusively on chronic pain and psychological approaches to managing symptoms such as cognitive behavioural therapy (CBT).^26–29^ Although results are promising,^26–29^ sample sizes are small and control groups are commonly either waiting lists or no treatment conditions, thus it is unclear how internet interventions when added to usual primary care might compare to usual primary care alone. GPs see the full spectrum of patients with LBP from acute to chronic/persistent pain. There is a need to explore the utility of internet interventions that have been designed to be broadly applicable for a wide range of patients presenting with LBP. It is also necessary to examine the provision of health professional support for LBP interventions with wider scope. CBT therapists have often been used to provide support for internet interventions specifically for chronic pain.^26^ Physiotherapists are integral members of the LBP clinical pathway in the UK NHS, and are well placed to deliver first-line rehabilitation with specialist skills in assessment and re-enablement to promote active and healthy lifestyles. Thus, physiotherapists may be the most appropriate choice to provide support for a LBP internet intervention targeting primary care patients.^30^

### Aims and objectives

We aim to explore the feasibility of providing an internet intervention for patients with LBP in primary care, with and without physiotherapist telephone support (in addition to usual care), compared with usual care alone. The feasibility of physiotherapists delivering limited telephone support for the intervention will also be explored.

Our objectives are to determine: (1) the feasibility of the trial procedures, including: recruitment of general practices and patients; the appropriateness of screening procedure and the selected measures; patient retention and data completion rates. (2) The acceptability of the internet intervention in supporting patient's use of, and adherence to, the intervention as well as physical activity recommendations, and the experience of patients in the intervention groups (this will be explored qualitatively). (3) The feasibility of physiotherapist telephone support, including: ability to contact patients; physiotherapists’ adherence to protocol; duration and number of calls made to patients. These objectives should enable decisions about the feasibility of moving to a definitive trial in future, and identify areas where the trial design may need to be modified.

## Methodology

### Design and setting

A single-blind, single-centre randomised controlled trial with three parallel arms to explore the feasibility of an internet intervention for patients with LBP in primary care. The trial will be carried out in 10–15 UK general practices in the south of England.

### Participants

The following eligibility criteria will be applied:

*Inclusion*: Patients will: have current LBP (have experienced pain within the past 2 weeks); have access to the internet and an active email address; have had a prior LBP consultation at their general practice within the past 6 months for those invited directly from practice lists; be able to read/understand English without assistance.

*Exclusion*: Patients will be excluded if: they are under 18 years of age; have clinical indicators of potentially serious spinal pathology or systemic illness; they have taken part in the study to develop the internet intervention is being trialled.

### Identification, invitation, screening and recruitment

This trial is supported by the UK Clinical Research Network Division 5 who are facilitating the recruitment of primary care practices for the trial. Once recruited, GP practice staff identity patients via two methods:

(1) Patients who have consulted with LBP in the past 3 months are identified from practice computerised records of consultations. The timeframe of the search will be extended to 6 months if the number of patients identified is limited. Lists of patients identified by the search are being screened by a practice GP who will rule out patients based on aspects of the eligibility criteria that can be determined from patient notes. Practices then send out study packs to the patients deemed eligible at this stage. Study packs include an invitation letter from the GP, a patient information sheet and contact details of the research team should the patient be interested in taking part. This method of identification and invitation of eligible patients is expected to be the main method used by practices.

(2) Patients are opportunistically identified by GPs in clinics. GPs briefly mention the study to eligible patients in the consultation and provide the patients with a study pack.

Patients who respond to the research team are further screened by the study manager. The study manager contacts the patient by phone and confirms eligibility by asking if they have current back pain (currently in pain, or have experienced pain in the past 2 weeks). Patients are then asked if they are experiencing 1 or more of 12 symptoms indicative of serious spinal pathology or systemic illness. These questions were initially developed for telephone-based musculoskeletal triage, and modified for this trial by the clinicians in the research team. See table 1 below for details.

**Table 1:** Screening questions relating to serious spinal pathology or systemic illness

High-level musculoskeletal safety questions			
I now need to ask you some very important questions			
1	Since this problem in your back started have you had a sudden, severe, worsening pain in the middle of your abdomen and upper back?	Yes	No
2	Do you have a new or recent problem in being able to pass urine?	Yes	No
3	Do you have a new or recent loss of control of your bladder and/or your bowels?	Yes	No
3a	Has your bladder been working properly? Can you tell when it is full? Have you felt that you want to ‘go’ all the time?	Yes	No
4	Do you have numbness or altered feeling or pins/needles around your back passage or genitals, for example, wiping after being at the toilet?	Yes	No
5	Since this problem in your back started have you had any heat, swelling or tenderness in your calf?	Yes	No
6	Since this problem in your back started have you been unable to put any weight at all through your legs?	Yes	No
7	Do you have constant pain that worsens at night?	Yes	No
8	Do you have a history of cancer?	Yes	No
9	Since this problem in your back started have you had any widespread or worsening weakness in your legs?	Yes	No
10	Did this episode of pain start following a fall, or get much worse and stay bad following a fall?	Yes	No
11	Do you have any chills or fevers or feel like you are running a temperature? (without other explanation)	Yes	No
12	Do you have trouble with your balance while walking?	Yes	No
12a	If ‘Yes’ above, has this changed during the current episode?	Yes	No

If a patient answers yes to one or more of these screening questions, the details are discussed with a senior clinician and where appropriate, referred back to their GP. If patients report that they are experiencing LBP, report no indicators of potential serious spinal pathology or systemic illness, and are interested in participating, they are emailed a link to the trial website. On this website, they provide online consent (patients have a chance to ask questions about the study in the screening phone call), complete online baseline measures and are automatically randomised to one of three intervention arms. If patients experience any technical difficulties during this online automated process, they receive telephone support from the study manager.

### Randomisation and blinding

**Figure 1:**
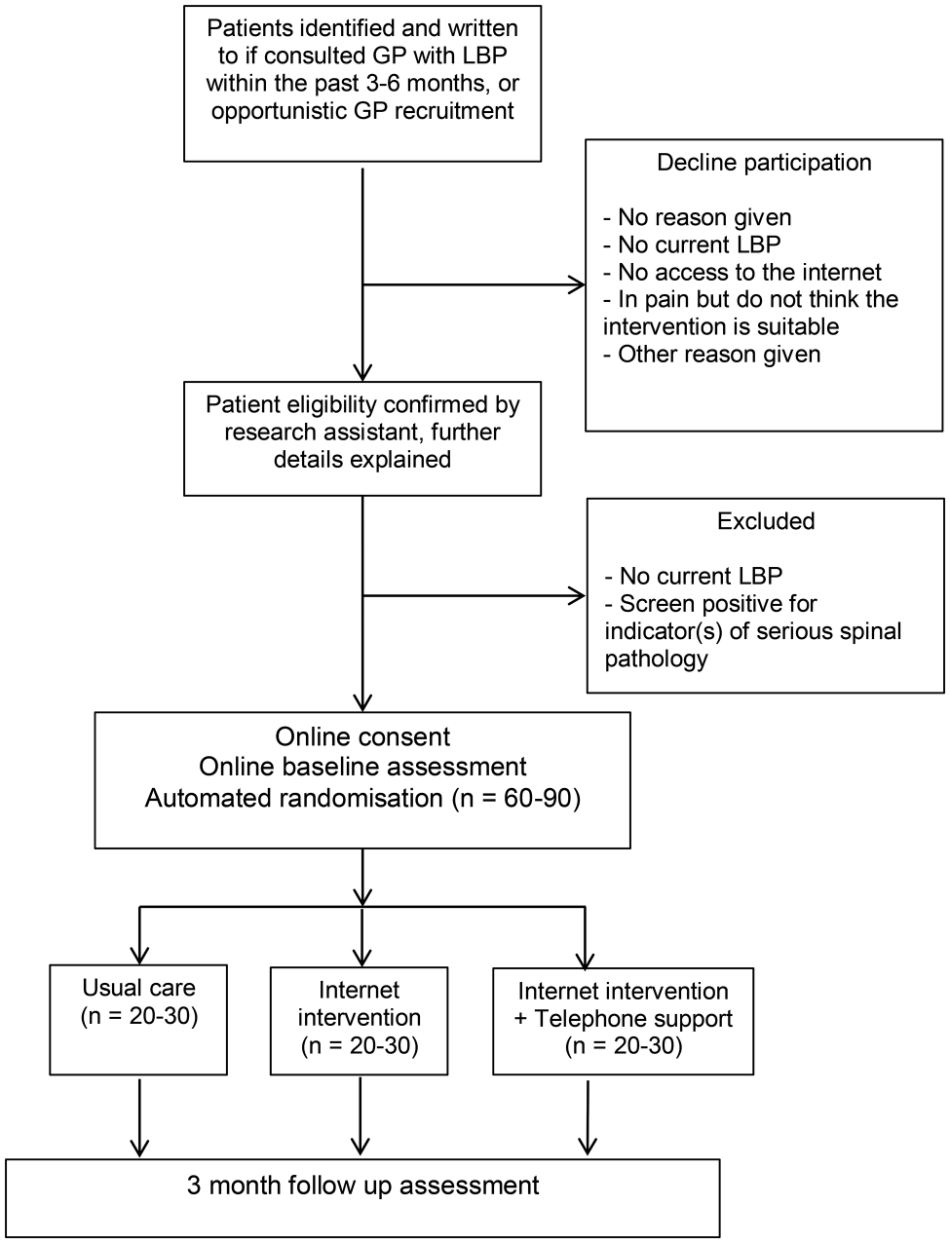
Diagram showing the flow of patients through the trial (GP, general practitioner; LBP, low back pain).

The internet intervention software will automate the randomisation process for this trial. The randomisation sequence is automatically generated, and a computer-generated algorithm block randomises patients to the trial groups. As the sample size is small, blocking is being used to increase the chance of approximately equal numbers of patients allocated to each group. Patients are stratified by severity (≥7 on the Roland-Morris Disability Questionnaire^31^). As the software randomises patients, the sequencing will be concealed from the trial team. Patients will be automatically informed of their allocated group via the internet through the intervention website. As the intervention is primarily behavioural, patients will not be blind to allocation. Telephone outcome data will be collected by an independent research assistant blind to group allocation. The trial statistician will remain blind to allocation until full analysis is finalised. See figure 1 for patient flow through the trial.

### Interventions

Details of the interventions (adhering to SPIRIT^32^ and TIDIER^33^ guidelines on reporting) are provided below.

#### Usual care

Patients allocated to this arm will continue to receive usual care from their GP. In the first instance, National Institute for Health and Care Excellence (NICE)^13^ recommended care for LBP consists of education and self-management advice, including advice to stay active. GPs may also make referrals to other health professionals who can offer other recommended treatments such as exercise programmes, manual therapy or acupuncture or to pain management programmes. With regard to pharmacotherapy, recommendations are for paracetamol initially, followed by NSAIDs or weak opioids.^i^ In practice, many GPs do not adhere to guidelines for LBP.^34^ It is likely that treatment received as part of usual care will vary, and this variation will be documented in the present study. If a patient does not reconsult over the study period, they may receive no additional care beyond that which they received as part of their initial GP consultation, whereas some patients may receive ongoing care from the GP, and/or referrals for diagnostic tests or treatments from other healthcare professionals such as physiotherapists or other specialists.

#### Usual care plus internet intervention

All patients in this arm will continue to receive usual care as specified above and will also have access to the internet intervention. ‘SupportBack’ was designed as an interactive multisession internet intervention, with the primary aim of providing patients with accessible information, tools and support to enable them to effectively manage their LBP. Internet provision allows the material to be accessed, and the suggested activities to be carried out wherever is most convenient for the patients. The intervention was developed using the open source LifeGuide software (http://www.lifeguideonline.org). The core of the intervention is focused on self-regulatory processes including graded goal setting, self-monitoring and tailored feedback to encourage physical activity/exercise increases or maintenance^35^ (see table 2 for more details of intervention content). The intervention also forms a substantial repository of educational advice regarding pain and LBP-related topics. Throughout, the included educational information has a focus on motivating behaviour change through techniques such as reassuring patients about likely consequences of movement; helping patients interpret mild pain; modelling through patient stories; reinforcing positive behaviour (using automated feedback); and providing simple instructions/demonstrations regarding how to perform various back-specific exercises/behaviours. By combining the above features with in-depth feedback from patients with LBP, the aim was to develop a highly accessible intervention that would support improvements in self-efficacy and have the potential to improve physical function.

**Table 2:** Summary table of optional SupportBack module content

Session number	Content
Session 1	How SupportBack works How SupportBack differs from other back pain websites Why activity is helpful for back pain, including: Reassurance Information about positive health consequences Commonly asked questions and responses regarding being more active while experiencing back pain Setting of activity goals: walking or back-specific exercises tailored to current functioning level Access to rationales, videos and benefits of activities Free to chose and amend activities Set goal level within tailored suggestions
Session 2 onwards	Goal review Feedback based on goal achievement and function level Opportunity to select new goals or keep the same Encouraged to select one module from the below menu A new module is available to select each session Patients can access their goals and selected module between sessions
**Additional modules**	**Content**
Sleep	Stretching before bed Sleeping positions Sleep checklist to improve sleep hygiene
Relieving pain	Pain medication Hot and cold therapy Everyday advice
Flare ups	‘First aid’ exercises Taking pain killers Better posture Alternative ways of easing pain
Work	Getting support from your employer Taking breaks Exercises to try at work Choosing a good chair Making your desk back friendly Using a laptop
Mood	Overview of mood and its connection to pain Cognitive behaviour therapy and mindfulness techniques for improving mood including: Self-kindness Increasing pleasant activities Mindful walking 3 min breathing space
Daily living	Sitting, standing and bending Lifting and carrying Shopping Doing housework In the bedroom In the bathroom In the kitchen Gardening

A person-based approach (PBA) was taken to the development process.^36^ This approach requires in-depth qualitative research to be a central component of intervention development. The aim is to ensure evidence-based suggestions and behaviour change theory are applied in such a way that the intervention fosters autonomy and is accessible and engaging for the target patient group.^36^ Twenty-two individuals, including 15 patients with LBP recruited from primary care practices and 7 from a local branch of the UK charity BackCare (formerly The National Back Pain Association), took part in interviews, through which they provided detailed qualitative feedback on an early pilot version of the internet intervention. Importantly, in line with the PBA, as well as providing comments on aesthetics and structure/navigation, participants were encouraged to discuss and provide detailed feedback on the content/advice based on their own lived experience of LBP. Elements of the content could then be reframed, or adjusted to ensure messages were not misconstrued. This method also allowed the identification of any areas where participants felt the intervention was ‘stating the obvious’ and then amend content, as far as possible, adding unique aspects to encourage engagement (see table 2 for further details).

The resulting internet intervention comprises six sessions. Patients are encouraged to access one session per week, to allow them to engage between sessions with the activity goals they have set themselves. Specifically, in the first session, patients are provided with information on how SupportBack will work, including the key rationale underlying the intervention; that keeping active is of primary importance when managing LBP. Likely concerns/potential barriers regarding this primary message are also addressed. The intervention then suggests two forms of activity patients can be supported with each week: walking or gentle back-specific exercises. Patients select one and set goals for the coming week. The recommendations provided are tailored, based on the extent patients report their LBP is obstructing their ability to engage with activities in their day-to-day lives.

From session 2 onwards, the intervention follows the same format. Patients review their goals from the previous week and are provided with automated tailored feedback and encouragement. They then have the opportunity to amend their goals, increase difficulty or switch to different activities. From session 2, after a patient's goal review, they can choose to explore one of six modules containing information and advice on a LBP-related topic (see table 2 for details). Exploration of these information modules becomes part of each broader ‘session’. Although patients are advised to work through a session per week, they can view a new session every 3 days if they wish. If engaged with as recommended, the intervention would take 6 weeks to complete. After the 6 weeks of structured sessions, patients will still have access to activity information and LBP-related modules as a static website. The intervention is fully automated and adherence is encouraged through weekly reminder emails containing links back to the intervention. A full development paper will be published describing the process of development in detail.

#### Usual care plus internet intervention plus physiotherapist telephone support

All patients in this arm also continue to receive usual care as specified above. The SupportBack internet intervention is offered to these patients with the addition of up to 1 h of telephone support from a physiotherapist. Trial physiotherapists are experienced musculoskeletal practitioners with spinal expertise. Although support will vary with patient need, it will not exceed 1 h in total but could be less and consists of 1 up to 30 min phone call followed by 2 up to 15 min phone follow-ups over 6 weeks. The purpose of the physiotherapy telephone contact is to provide support and encouragement for use of the internet intervention, and to address any concerns in relation to the internet-based content. The physiotherapists are asked to closely adhere to a standardised content checklist for each phone call (available on request to the corresponding author). While they are able to address individual patient concerns, they are asked to avoid additional individualised patient assessment and treatment recommendations beyond the internet intervention content to adhere to protocol fidelity.

Call 1 (up to 30 min) is planned to take place between weeks 1 and 2 after randomisation. In this call, the physiotherapist explores and addresses the patient's understanding and attitudes (eg, belief that activity can be helpful for back pain); engagement with the internet intervention (eg, enquiring how patient has got on with their goals); anticipating barriers (asking what problems patients anticipate in participating in the SupportBack programme). Calls 2 and 3 (up to 15 min) are planned to take place between weeks 2 and 3, and between weeks 4 and 5. In these telephone calls, the physiotherapist discusses general adherence; congratulates any adherence behaviour to both the internet intervention and physical activity goals; discusses barriers to adherence and how these might be addressed; encourages commitment to goals for the following week; and addresses any remaining concerns.

### Intervention fidelity assessment

Use of an automated internet intervention will ensure all patients in the intervention arms have access to the same information and advice delivered through this medium. A 2 h training session was provided for trial physiotherapists, along with detailed checklists of topics to be covered in each telephone call in order to attempt to maintain treatment fidelity in the telephone support arm. Intervention fidelity will be assessed in this arm by recording all telephone sessions and examining any systematic deviations from the protocol.

### Outcomes and measures

#### Primary feasibility outcomes

The primary outcomes from this feasibility trial are descriptive, relating to the number of practices required to recruit the patient numbers; whether it is possible to recruit the target number of patients and in what time frame, including eligibility and recruitment rate per practice per month; whether problems were encountered with the screening of patients; number of patients withdrawing from the interventions and the trial and follow-up retention rates at 3 months. With regard to the internet intervention, the extent of patients’ usage will be described. The process of physiotherapist support will also be described, along with adherence to the protocol and any significant issues encountered.

#### Additional measures

Demographic data including gender, age, education, occupation and marital status are collected at baseline. See table 3 for full details of measures included. A range of LBP-related measures will be explored including disability, pain, pain-related psychological processes (eg, catastrophising and kinesiophobia), quality of life as well as physical activity. The clinical measures selected align with recommended outcome domains for clinical trials targeting LBP such as physical functioning, pain intensity and health-related quality of life.^37^ Measures are also included to inform future health economic analysis, for example, health resource use, examine adherence to back-related activities, and explore potential predictors that may affect engagement with the internet interventions (eg, exercise self-efficacy, credibility and expectancy). Patient adherence to the internet intervention will be explored by examining objective intervention usage data automatically collected by the LifeGuide internet intervention. These data will provide detailed information on number of logins, number of sessions accessed, physical activity goals set, module accessed as well as time spent on each webpage.

**Table 3:** Variables, measures and their characteristics

Variable	Measure	Items	Details	Reliability where available	Administration point, trial arms
Pain duration	Pain duration^38^	1	This single item asks ‘how long it was since you had a whole month without pain’. Response options range from ‘less than 3 months’ to ‘over 10 years’^38^		Baseline All arms
Back-specific physical disability	RMDQ^31^	24	Patients select from a list of items that may describe their experience on the day of scale completion (eg, ‘I sleep less well on my back’). The scale is dichotomous, patients select whether the symptom is present or not	Internal consistency: 0.77–0.93^39^	Baseline, follow-up All arms
Pain intensity	Pain index created by three numerical rating scales^40^	3	Patients rate their current pain, average and least pain over the past 2 weeks on three 11-point rating scales	Test-retest reliability: 0.67–0.96^41^	Baseline, follow-up All arms
Days in pain	Number of troublesome days in pain over the last month	1	Based on a measure used by Little *et al*,^42^ this single item asks patients to record the number of troublesome days they have spent in pain over the last month		Baseline, follow-up All arms
Risk of persistent disability	SBST^40^	9	Patents indicate whether they disagree or agree with items covering modifiable prognostic indicators including bothersomeness, disability and mood. Patients are asked to respond thinking about their past 2 weeks	Internal consistency: 0.79^40^	Baseline, follow-up All arms
Fear of movement	TSK^43^	17	Patients rate agreement with items including ‘I'm afraid I will injure myself if I exercise’. Items are scored from 1 to 4, ranging from strongly disagree to strongly agree	Internal consistency range: 0.70–0.79^44^	Baseline, follow-up All arms
Negative orientation toward pain	PCS^45^	13	Patients indicate their agreement with items following the stem ‘When I'm in pain…’ Items include ‘I feel like I can't go on’ and are scored on a scale of five-point scale ranging from ‘not at all’ to ‘all of the time’	Internal consistency: 0.81^46^	Baseline, follow-up All arms
Physical activity	Short form IPAQ^47^	7	Patients provide time spent walking, undertaking vigorous and moderate activity, as well as time spent sedentary over the past 7 days	Test-retest reliability range: 0.65–0.88^47^	Baseline, follow-up All arms
Enablement coping/satisfaction	Modified PEI^48^	6	Patients are asked to consider the healthcare they have received in the past 3 months and rate items including ‘I am able to cope better with my back problem’ and ‘I am able to understand my back problem better’. Items are scored on a seven-point scale from strongly agree to strongly disagree	Internal consistency: 0.92^48^	Baseline, follow-up All arms
Quality of life	EuroQol EQ-5D^49^	6	The EQ-5D consists of two parts. Part 1 consists items on five domains, for example, mobility, self-care. Part 2 features a VAS that records patient's ratings of overall health		Baseline, follow-up All arms
LBP-related health care resource use	Measured with a brief questionnaire developed for this trial	10	LBP resource use measured will include medication use, number of GP visits, other NHS care and private LBP related-therapy costs		Follow-up All arms
Time off work	Measured with a single item developed for this trial	1	Patients are asked how much time they have had off work in the past 3 months		Follow-up All arms
Beliefs about effectiveness and credibility of the interventions	Modified CEQ^50^ ^51^	6 2	Patients rate the extent they think/ feel the intervention is likely to improve their back pain-related functioning, and credibility. Items range from 1 not at all to 9 very much. Two items measuring how patients think and feel about likely improvement are measured as a percentage. Two items drawn from the expectancy section of the questionnaire will be asked to all patients	Internal consistency range: 0.82–0.84^50^	Following session 1 Internet intervention arms only Baseline All arms
Exercise self-efficacy	Modified Self-Efficacy for Exercise scale^52^ ^53^	8	Patients rate how confident they are on a scale between 0 and 10 that they could complete activities suggested by the internet intervention in the face of obstacles such as ‘if they felt pain’, ‘if they were bored’ and ‘if they were depressed’	Internal consistency: 0.92^53^	Following session 1 Internet intervention arms only
Reasons for non-adherence	PETS^54^	18	The scale is designed specifically to measure reasons for patient non-adherence to home-based rehabilitation. Patents rate their agreement with items on a five-point scale across domains including problems with symptoms, uncertainty, doubts, practicalities and lack of support	Internal consistency for the PETS subscales range: 0.84–0.96^54^	Follow-up All arms
Adherence to specific activities for LBP	Items developed specifically for this study	4	Patients are asked about the number of weeks if any they may have walked and/or engaged gentle back exercises. They are also asked estimate of how many days a week they went for walks and/or did gentle back exercises. Patients are also asked if they stopped activities because they no longer are experiencing pain		Follow-up All arms

CEQ, Credibility and Expectancy Questionnaire; GP, general practitioner; IPAQ, International Physical Activity Questionnaire; LBP, low back pain; NHS, National Health Service; PCS, Pain Catastrophising Scale; PEI, Patient Enablement Instrument; PETS, Problematic Experiences of Therapy Scale; RMDQ; Roland-Morris Disability Questionnaire; SBST, STarT Back Screening Tool; TSK, The Tampa Scale for Kinesiophobia; VAS, Visual Analogue Scale.

A GP notes review will be conducted to determine LBP-specific GP consultations and referrals over the trial period. The aim will not be to carry out a detailed costing study but to identify main drivers of cost to inform the future trial and to determine the feasibility of collecting health economic measures from primary care practices.

### Nested qualitative components

Nested qualitative work will enable patients’ experiences of the trial and allocated arms to explore inductively. This component was designed to allow thematic analysis as outlined by Braun and Clarke.^55^ Approximately 20 patients will be recruited to take part in semistructured telephone interviews. Patients across all three arms of the trial will be purposively sampled to ensure diversity in terms of age, gender and clinical profile (functioning, pain severity/duration). Patients in the internet intervention arms will be also be sampled based on high and low usage of the internet intervention. The aim of the interviews will be to explore patients’ experience of taking part in the trial including difficulties, positive elements and other salient issues. For those patients in internet intervention groups, their experience of the programme and telephone support will be explored in detail. The same sampling frame will be used to explore the content of telephone support sessions for patients in that arm.

### Sample size

Guidance on appropriate sample size for feasibility trials varies with suggestions from n=12 to n=30 plus per arm.^56^ ^57^ Working within the resource restrictions for this trial, we aim to recruit between 60 and 90 patients, approximately 20–30 patients allocated to each of the three arms. A sample of this size will enable feasibility objectives to be met (including exploration of patient recruitment, adherence and retention) as well as enabling initial estimates of variability in key quantitative outcomes. This sample size also allows sufficient patients to draw from in order to undertake a nested qualitative study.

### Data collection, storage and sharing

LifeGuide is online software that integrates an intervention platform with a data management system. Primarily, LifeGuide will be used to collect all data. Data collected during the intervention period will include logins, page views and time spent on each page. Patients in all trial arms will complete baseline measures online. They will be automatically emailed at 3 months and asked to complete follow-up measures online. Failure to respond to two emails and a written letter will lead to the patient being called by an independent research assistant, blind to treatment allocation, who will collect the key disability and pain-related quantitative outcomes over the telephone. After 3 months, all patients who were randomised will have their medical notes reviewed and consultations and LBP-related resource use recorded in order to inform the feasibility of a cost-effectiveness analysis in the future full trial. All patient data are being kept in strict confidence and managed in accordance with the Data Protection Act 1998. The University of Southampton policy on archiving will be followed; the data will be stored for 10 years following the end of the study, after which time it will be disposed of securely. Following completion of the trial, a cleaned anonymised data set will be shared on request.

## Analysis

### Quantitative analysis

The primary quantitative analysis will focus on a description of feasibility outcomes. The number of practices recruited, patient eligibility and recruitment rates will be presented. We will also present the number of withdrawals from the trial per arm, along with numbers/percentages of dropouts from the intervention who do not respond to follow-up. Use of the internet intervention will be described by presenting automated data collected on frequency of logins and time spent on the intervention for both the stand alone intervention and the intervention plus telephone support arm. With regard to the internet intervention plus telephone support arm, the number of support calls successfully made, along with the mean number per patient in this arm will be described.

Exploratory analysis of patients’ clinical outcome (eg, disability/pain) and scores on additional measures will be conducted. The completeness of the measures, along with any floor or ceiling effects will be described to inform selection of outcomes and measures for a future definitive trial. Descriptive statistics will be used to provide means, SDs and CIs for the measures. Correlations between baseline data including demographics, severity disability/pain, pain duration and website usage/trial attrition will be examined. This may provide an initial indication of potential relationships between patient factors, an engagement with the internet intervention and the trial procedures.

Effectiveness data would be required to perform an economic evaluation; nonetheless, in this feasibility study, we will collect resource use and outcomes data to inform the design of the definitive trial. Self-reported healthcare usage will be compared with consultations and NHS healthcare use recorded in patient clinical notes. Where similar data are collected, for example, GP visits, the comparison will enable us to make a judgement on the comparative performance of the two measures and will inform the choice of measure for the definitive study. Any difficulties practices have with completing the notes review forms will be recorded, and processes amended for the main trial. The EQ-5D will be used for the preliminary calculation of quality adjusted life years

### Qualitative analysis

Thematic analysis^55^ will be used to analyse resulting transcriptions from patient interviews and will be conducted in phases; (1) initial codes will be generated; (2) themes will be identified; (3) all themes will be reviewed; (4) themes will be defined and named; (5) final analysis including interpretation and write up. An audit trail will be kept and both coding and interpretation will be cross-checked among the research team. The full process will be iterative using discussion and reflective feedback to identify discrepancies in the analytic process. A thematic analysis will also be conducted using transcriptions of initial physiotherapist-patient telephone calls in order to inductively explore key issues discussed in telephone sessions.

### Success criteria

The feasibility of the trial and intervention will be assessed based on the following criteria:

- Recruiting a minimum of 60 patients with LBP, access to the internet and without indicators of serious spinal pathology from primary care within the allotted recruitment time period for the trial.
- Attrition at 3-month follow-up should be equal to or lower than 30% from all trial arms.
- By examining the recordings, the telephone support physiotherapists are able to deliver the telephone sessions in line with the protocol, covering approximately 2/3 of the checklist in each call.
- Patients should be able to access the intervention and complete measures, complete session 1 and set goals for future sessions. This will be assessed by examining LifeGuide software collected usage data.
- Qualitative and quantitative data should indicate that the intervention and trial procedures are acceptable to patients.

### Harms/adverse events

The risk of adverse events occurring as a consequence of the interventions in this trial is low. All activities recommended (walking and back-specific exercises) are explained in detail with an emphasis on choosing a level that is comfortable for the patient, and amending quickly if the patient feels the initial level is too high. Nonetheless, adverse events will be recorded and reported to the trial team. Where necessary, the event will be discussed with clinical members of team, and the patient will be referred to their GP. Serious adverse events that do occur will be recorded and reported to the ethics committee.

## Ethics and dissemination

All patients are provided with detailed information about the trial and have a chance to discuss procedures with a member of the study team before consenting to take part. Patients will be asked to give their consent to take part in the study, to have their medical records accessed, to have the data they have provided used unless they refuse, and to take part in a nested qualitative component of the study following the end of the intervention period. All patients are clearly informed that they can withdraw from the trial at any point.

As this is a feasibility trial, the main focus on dissemination would come after a future definitive trial. Nonetheless, the aim will be to publish our feasibility results in leading academic journals, and present the research at national and international primary care and musculoskeletal conferences.

## Discussion

Internet interventions have many characteristics (automation, tailoring, readily accessible), that when co-developed with target users, have the potential to help primary care patients manage their symptoms. However, this potential must be explored rigorously through fully powered effectiveness trials. The present feasibility trial represents a necessary step along that path. If feasibility is demonstrated, the trial design will be refined where needed and the aim will be to move to a full-scale definitive study of the SupportBack intervention.

## Footnotes

i The full guideline can be found here: https://www.nice.org.uk/guidance/cg88.
